# E-Skin Bimodal Sensors for Robotics and Prosthesis Using PDMS Molds Engraved by Laser †

**DOI:** 10.3390/s19040899

**Published:** 2019-02-21

**Authors:** Andreia dos Santos, Nuno Pinela, Pedro Alves, Rodrigo Santos, Ricardo Farinha, Elvira Fortunato, Rodrigo Martins, Hugo Águas, Rui Igreja

**Affiliations:** CENIMAT|i3N, Departamento de Ciência dos Materiais, Faculdade de Ciências e Tecnologia, Universidade Nova de Lisboa, Campus de Caparica, 2829-516 Caparica, Portugal; ass.santos@campus.fct.unl.pt (A.d.S.); n.pinela@campus.fct.unl.pt (N.P.); pedro.alves@strath.ac.uk (P.A.); rcd.santos@campus.fct.unl.pt (R.S.); rd.farinha@campus.fct.unl.pt (R.F.); emf@fct.unl.pt (E.F.); rm@uninova.pt (R.M.); hma@fct.unl.pt (H.Á.)

**Keywords:** electronic skin, pressure, piezoresistivity, micro-structuration, temperature, laser engraving, molds, bimodal sensors, robotics, prosthesis

## Abstract

Electronic skin (e-skin) is pursued as a key component in robotics and prosthesis to confer them sensing properties that mimic human skin. For pressure monitoring, a great emphasis on piezoresistive sensors was registered due to the simplicity of sensor design and readout mechanism. For higher sensitivity, films composing these sensors may be micro-structured, usually by expensive photolithography techniques or low-cost and low-customizable molds. Sensors commonly present different sensitivities in different pressure ranges, which should be avoided in robotics and prosthesis applications. The combination of pressure sensing and temperature is also relevant for the field and has room for improvement. This work proposes an alternative approach for film micro-structuration based on the production of highly customizable and low-cost molds through laser engraving. These bimodal e-skin piezoresistive and temperature sensors could achieve a stable sensitivity of −6.4 × 10^−3^ kPa^−1^ from 1.6 kPa to 100 kPa, with a very robust and reproducible performance over 27,500 cycles of objects grasping and releasing and an exceptionally high temperature coefficient of resistance (TCR) of 8.3%/°C. These results point toward the versatility and high benefit/cost ratio of the laser engraving technique to produce sensors with a suitable performance for robotics and functional prosthesis.

## 1. Introduction

Nature is an endless source of inspiration for the human being. Almost everything that surrounds us may be looked at with a different perspective, seeking new ideas to improve our lives. Human skin is often underrated due to the simplistic way people look at it: an organ that protects other organs that are considered to be vital for our existence. However, if one starts considering all the functions attributed to skin, its complexity immediately acquires a dimension previously unrecognized; skin not only protects us from pathogenic agents and other environmental aggressions like ultraviolet (UV) light, but also plays an important role for the homeostasis of the human body, being also equipped with several biological sensors to detect temperature, humidity, pain, mechanical forces, textures, and so on [1,2]. The creation of a human skin surrogate with some or all the mentioned sensing properties, as well as flexibility and stretchability, the so-called electronic skin (e-skin) [1], is pursued by the scientific community aiming for its application in a variety of fields, from robots [3,4,5] to health monitoring [6,7,8,9,10,11,12,13,14,15] and functional prosthesis [16,17,18]. 

Sensors dedicated to pressure sensing may rely on some distinct effects to accurately transduce a pressure signal into an electrical signal, namely piezoresistivity [19,20,21,22,23,24,25,26,27], capacitance [6,17,28,29,30], piezoelectricity [8,15,31,32], and triboelectricity [33,34,35,36]. Given that it is quite simple to both design and read the output signal of a sensor that plays on piezoresistivity, this effect fueled a great level of research in the field, where a pressure variation is transduced into a change in resistance, mostly derived from changes in the geometry of the sensing element, the resistivity of a composite due to changes in separation between particles, or the contact resistance [1]. For the latter, the creation of micro-structured domains in films that compose the sensors increases the contact area between films, thereby increasing the R_OFF_/R_ON_ ratio [9,23,27] and, consequently, translating into an improved sensor sensitivity.

The majority of sensors so far reported typically exhibit two different sensitivity values depending on the applied pressure: a higher value for low pressures and a significantly lower value for higher pressures [6,9,20,22,23,24,25,26,28,30,34,37,38,39,40,41]. If such sensors are to be used in the wrist for the detection of the blood pressure wave, the requirement is for these to show a high sensitivity below 400 Pa [19]. In the case of functional prosthesis and robotics, the need is different; sensors should be able to accurately detect pressure stimuli in a range that is associated to human common interactions with the surroundings, from less than 10 kPa for gentle touch to 100 kPa for object manipulation [20,28]; thus, a constant sensitivity in such a pressure range would be preferable. Moreover, the two most explored approaches for the sensor micro-structuration are based on the use of either expensive and time-consuming photolithography techniques [6,9,10,11,12,19,23,26,27,28,33,34,35,37] or low-cost but also low-customizable techniques consisting of the use of everyday objects as molds [22,36,42]. There is, therefore, a need for the development of a low-cost e-skin pressure sensor for which production is based on simple and highly customizable techniques. Such customization is crucial to tailor the performance of the sensors to each application, either showing a high sensitivity in a low-pressure range for health applications, or a constant sensitivity in a wide pressure range that is meaningful for prosthesis and robotics applications. 

In order to sense both pressure and temperature, researchers worked either on sensors able to simultaneously detect both stimuli or on the integration of multiple sensors. While the first option may imply complex data analysis to distinguish the contribution of each stimulus to the sensor’s output [43,44], the second requires special attention to prevent or minimize sensors’ cross-sensitivity and may lead to bulky sensors [16,45], which still leaves room for further research and improvement of current e-skin sensors to achieve more functional prosthesis and robots.

This group recently introduced a micro-structuration technique for polymeric films based on the easy and fast production of highly customizable acrylic molds through a laser engraving equipment [46]. The resultant sensors, with micro-cones as their micro-structuration, exhibited a high sensitivity of −2.5 kPa^−1^ below 160 Pa, a value that decreases for higher pressures. Herein, other designs for the molds were explored in order to tailor the sensitivity of the sensors and extend the correspondent pressure range from less than 10 kPa to 100 kPa to make them suitable for prosthesis and robotics applications. Instead of using acrylic, hard polydimethylsiloxane (h-PDMS) was explored as the material of the molds. With circular cavities engraved in the molds, standard PDMS (s-PDMS) films with semi-sphere like structures were patterned by soft-lithography and used in e-skin piezoresistive sensors that could achieve a sensitivity as high as −6.4 × 10^−3^ kPa^−1^ between 1.6 kPa and 100 kPa, a pressure range that is relevant in the field of robotics and prosthesis. These sensors could also achieve a temperature coefficient of resistance (TCR) of 8.3%/°C. Poly(methyl methacrylate) (PMMA) was studied as an adhesion layer between carbon coating (the active material of the sensor) and s-PDMS. Sensors that had this layer presented a more stable performance and faster relaxation times until a minimum of 28 ms, which made them better than sensors without PMMA. Fatigue tests were conducted in these sensors by placing them in a robotic arm and evaluating their performance over 27,500 cycles of grasping and releasing of an object, proving their relevance for this type of application. Despite not exploring innovative shapes for the micro-structures nor a new design for the pressure and temperature sensors, this work, which is an extended version of a conference paper [47], ultimately shows the versatility of laser-engraved molds for the production of sensors suitable for a plethora of applications, where one only needs to change the design features of the sensor to have a substantial impact on its performance. Furthermore, although the micro-structures achieved through this technique are not as homogeneous and regular as those produced through photolithography techniques, they were proven to be very functional, which highlights the high benefit/cost ratio of using laser engraving for the micro-structuration of films for e-skin sensors. 

## 2. Materials and Methods

### 2.1. Chemicals and Materials

PDMS elastomer and curing agent (Sylgard 184) were purchased from Dow Corning (Midland, MI, USA). PMMA (molecular weight (MW) approximately 120,000) and trichloro(1*H*,1*H*,2*H*,2*H*-perfluorooctyl)silane (97%) were purchased from Aldrich (St. Louis, MO, USA). Toluene (99.99%) was purchased from Fisher Scientific (Loughborough, UK). Highly conductive water-based carbon coating (PE-C-808) and water-based silver conductive ink (PE-WB-1078) were purchased from Conductive Compounds (Hudson, NH, USA).

### 2.2. Fabrication and Laser Engraving of h-PDMS Molds

Patterns to be engraved in the molds were designed in Illustrator^®^ (2015.0.0) and exported as computer-aided design (CAD) files. These patterns consisted of a repetition of circles with a diameter of 100 µm or 200 µm and a pitch of 150 µm or 200 µm over an area of 2 cm × 2 cm.

**Figure 1:**
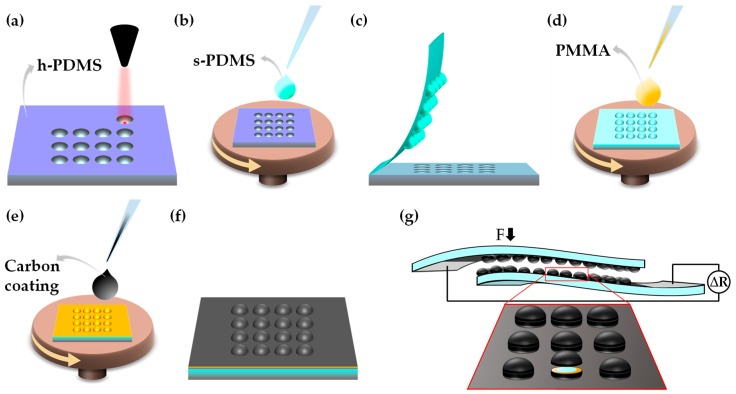
Fabrication process of an e-skin piezoresistive sensor. (**a**) Laser engraving cavities on a hard polydimethylsiloxane (h-PDMS) mold. (**b**) Standard PDMS (s-PDMS) spin-coating on the engraved mold. (**c**) Peeling off the flexible s-PDMS film from the mold. (**d**) Poly(methyl methacrylate) (PMMA) spin-coating on the s-PDMS film. (**e**) Carbon coating spin-coating on the previous film. (**f**) Final micro-structured film after curing of carbon coating. (**g**) Final device with a silver-ink stripe on the smooth edge of each film. None of the steps are to scale.

Hard PDMS molds were fabricated by mixing PDMS curing agent with PDMS elastomer in a 1:5 *w*/*w* ratio. The mixture was then degassed in vacuum for 30 min, poured into a Petri dish, and cured for 1 hour at 70 °C. The h-PDMS molds (5 mm thick) were micro-structured using a laser engraving machine (VLS3.50, 50 W, Universal Laser System, USA) with a CO_2_ laser beam, a lens focus length of 2.0 in, and a focal spot of 127 μm in diameter, according to the imported CAD file, as shown in Figure 1a. The engraving was performed with a laser speed of 0.254 m/s and a laser power of 2.5 W, 7.5 W, 12.5 W, or 25 W. 

The molds were afterward cleaned in an ultrasonic bath for 10 min in isopropanol alcohol, rinsed in MiliQ water, and dried with compressed air. Then, the molds were placed in a desiccator for 30 min, together with 1 drop of trichloro(1*H*,1*H*,2*H*,2*H*-perfluorooctyl)silane, to be covered by a hydrophobic layer and facilitate the peeling of s-PDMS films.

### 2.3. Fabrication of E-Skin Sensors

Standard PDMS was prepared in a ratio of 1:10 *w*/*w* of curing agent to elastomer and was subsequently degassed in vacuum for 30 min. The mixture was then spin-coated at 250 rpm for 90 s onto each engraved h-PDMS mold (Figure 1b), followed by another degassing process and curing at 85 °C for 30 min in an Infrared IC Heater (T-962 Eco-Worthy). Posteriorly, the micro-structured s-PDMS films were easily peeled off, as shown in Figure 1c, and submitted to an O_2_ plasma treatment (37.5 W for 1 min with an O_2_ pressure of 0.3 mbar) before spin-coating PMMA (10 wt.% in toluene) at 1000 rpm for 1 min (Figure 1d). Thermal curing of PMMA films occurred in vacuum for 1 h at 140 °C.

The PMMA-coated s-PDMS films underwent another O_2_ plasma treatment before spin-coating highly conductive water-based carbon coating (PE-C-808) at 1000 rpm for 20 s (Figure 1e). The carbon coating was cured for 30 min at 85 °C to finalize the treatment of each film, as shown in Figure 1f. After cutting the s-PDMS films (3 cm × 2 cm), pairs of films with the same pattern were sandwiched and sealed with s-PDMS. Lateral transducer electrodes were created by depositing lines (1 cm width) of 50 wt.% in water of water-based silver conductive ink (PE-WB-1078) on the edges of each film. The curing of this ink was done for 120 s at 145 °C. A scheme of a final e-skin piezoresistive sensor is shown in Figure 1g, with the inset highlighting the semi-spheres and all the layers that compose them. 

### 2.4. Morphological Characterization of Micro-Structured s-PDMS Films

Micro-structured h-PDMS molds and s-PDMS films were coated with a gold/palladium (Au/Pd) layer of 15 nm and observed with a tabletop scanning electron microscope (SEM) (Hitachi TM3030Plus) in standard observation mode at 15 kV. Measurements of the micro-features were performed using the software ImageJ (1.49b). A profilometer (Ambios XP-Plus 200 Stylus) using a tracking force of 1 mg and a speed of 0.10 mm∙s^−1^ was used to estimate the roughness of PDMS films, performing five to 10 measurements of each sample. A contact-angle equipment (Dataphysics OCA15plus) was used to measure the contact angle in PDMS films before and after the oxygen plasma treatment referred to in Section 2.3.

### 2.5. Electrical Characterization of Films and Sensors

A Keithley 617 Programmable Electrometer was employed for the acquisition of current–voltage (I–V) curves of the films and sensors, with a voltage sweep from −2 V to 2 V, in steps of 0.5 V. To test the sensors response to changes in pressure, several weights were loaded on the sensors to apply pressures from 15 Pa to 100 kPa, at a constant voltage of 5 V to 10 V. To estimate the relaxation time, a small magnet (~184 Pa) was loaded and unloaded on top of the sensors. The temperature response of the sensors was evaluated by applying a temperature in increments of 1 °C from 25 °C to 45 °C while monitoring the nominal resistance change. Pressure stability tests were conducted after mounting sensors in a robotic arm controlled by a servomotor. The sensors monitored the pressure exerted by the robotic arm during the grasping of a volumetric flask, which was performed 27,500 times. Except for the temperature response evaluation, all the electrical characterization of the sensors was performed at room temperature. The impact of humidity on the performance of the sensors was not evaluated, although all the characterization was performed at a constant humidity of 60%, and the sensors could be completely laterally sealed to avoid the contact of the carbon coating with atmospheric humidity.

## 3. Results and Discussion

### 3.1. Hard PDMS Molds Patterning

**Figure 2:**
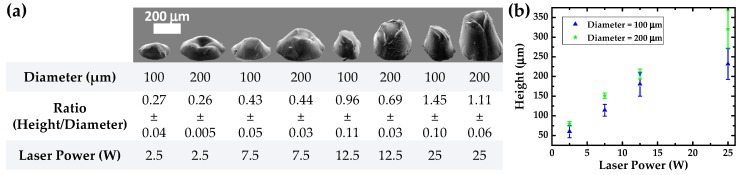
Effect of laser power and circle diameter on micro-structures peeled off from h-PDMS molds, with an engraving laser speed of 0.254 m/s and a circle pitch of 200 µm. (**a**) SEM images of micro-structures with the respective height/diameter ratio. (**b**) Micro-structure height variation with laser power and circle diameter. Note that the values correspond to average values ± standard deviation of a minimum of three measurements.

The interaction between the laser beam and PDMS was explored for the production of h-PDMS molds. When the CO_2_ laser hits h-PDMS, its radiation (10.6 µm) is greatly absorbed by the polymer [48], which will then heat locally and go through a photothermal evaporation process [49,50], resulting in the creation of cavities in the bulk material. In order to investigate the effect of laser power on some features of the molds’ micro-cavities, molds were engraved with a design based on circles with a pitch of 200 µm using a laser power of 2.5 W, 7.5 W, 12.5 W, or 25 W, while fixing the laser speed at 0.254 m/s. This high value of laser speed reduces the working time of the laser on h-PDMS and, thus, induces the production of smaller and less sharp micro-structures. The effects of this parameter, in addition to different circle diameters (which varied between 100 µm and 200 µm), were evaluated by checking the final features of s-PDMS films micro-structured through these engraved molds. Figure 2a,b present the results of this study, where one observes that the height of s-PDMS micro-structures increases with laser power, which is explained by the greater melting of h-PDMS in depth, thus creating deeper cavities that result in higher micro-structures. A relationship between diameter and height is also observable, whereby a larger designed diameter resulted in a higher semi-sphere. This results from the fact that the engraving of larger areas induces a greater h-PMDS melting in depth. The roughness of the smooth part of the micro-structured films was estimated to be less than 100 nm, without significant variations in the laser parameters.

Given that one of the main goals of the present work was to achieve an e-skin sensor with a stable sensitivity in a wide pressure range, the choice of the micro-structures of the films composing the sensor plays a critical role. As it was pointed out by the work of Choong and colleagues, micro-structures with a pyramidal shape are more compressible than pillars with a circular base; therefore, sensors with pyramids have a greater sensitivity for low pressures than sensor with pillars [23]. However, this sensitivity decreases for higher pressures, and this phenomenon is common to other works [34,46] due to the complete deformation of these highly compressible micro-structures. As a consequence of these studies, less compressible structures such as semi-spheres were developed, pursuing the production of piezoresistive sensors that are able to withstand larger pressures while maintaining a constant sensitivity, even if such a parameter is not maximized for low pressures. To achieve a micro-structure similar to a semi-sphere, not only should its shape be round, but the ratio of its height to its diameter should be close to 0.5. For a laser power of 12.5 W and 25 W, the referred ratio was much greater than 0.5, mainly because the micro-structures were higher. Regarding these results, a laser power of 7.5 W seems to be more appropriate to produce semi-sphere like structures, given that the height/diameter ratio for this condition was closer to 0.5. 

**Figure 3:**
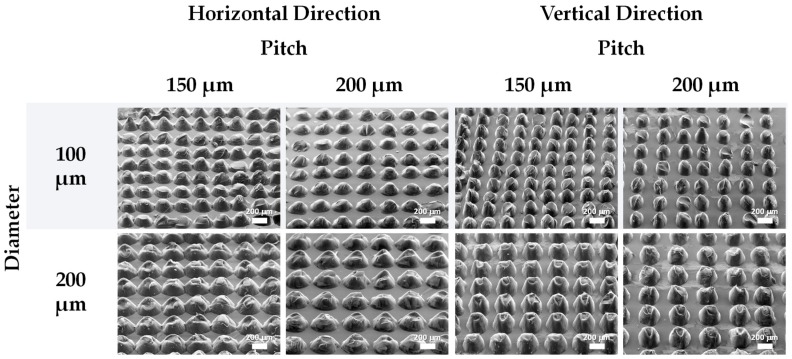
SEM images of s-PDMS micro-structured films peeled off from h-PDMS molds engraved with circles of different diameters and pitches, with a laser power of 7.5 W and a laser speed of 0.254 m/s. The first and second columns of images show the micro-structures according to a horizontal laser engraving direction, and the remaining columns of images show the micro-structures according to a vertical laser engraving direction.

Micro-structures resultant from two similar designs were used to compare the theoretical diameter and pitch with the real measurements of these features, as well as to compare the laser engraving resolution over vertical and horizontal directions. Such micro-structures were produced after engraving molds with circles with a theoretical diameter of 100 μm or 200 μm and a pitch of 150 μm or 200 μm, using a laser speed of 0.254 m/s and a laser power of 7.5 W. The general view of the resultant s-PDMS micro-structured films shows a nice homogeneity for each pattern while highlighting the differences between pitches and diameters over horizontal and vertical directions, as presented in Figure 3. Each feature can be easily discerned even for the lower pitch, meaning that a theoretical pitch of about 150 μm was achievable with this technique and with the laser parameters used. Local defects in the pattern may be attributed to the engraving of the molds or the peeling off process. Given the limitations of the laser equipment’s resolution, the laser may engrave some defects in the molds, which are then transmitted to the micro-structured films. The peeling off process may introduce additional defects to the micro-structures because there are some adhesion forces between the h-PDMS of the molds and the s-PDMS of the films. Although these forces are minimized through a silanization process that makes the molds more hydrophobic, they are still present and may hamper the peeling off of the films. Hence, pieces of s-PDMS may get stuck inside the cavities of the molds, giving rise to broken micro-structures seen in the SEM images of Figure 3.

The measurements for both diameter and pitch of the micro-structures are often far from the designed values; however, their sum tended to be very close to the sum of the designed features, as highlighted in Table 1. This can be explained by the fact that the laser beam melts more h-PDMS than designed, thus enlarging the diameter of the cavities. Therefore, the pitch is much smaller than expected to maintain the design. The laser engraving resolution in the vertical direction is notably better than in the horizontal direction, given that features measured in the vertical direction of the laser engraving show diameter and pitch values closer to the designed ones. Such dissimilarity may be attributed to either the low cost of the laser equipment (which is less precise than other more costly equipment) or an asymmetry of the beam spot, resultant from the distance between the laser source and the spot where the laser engraves the material, as observed by Fogarty and colleagues [49].

**Figure 4:**
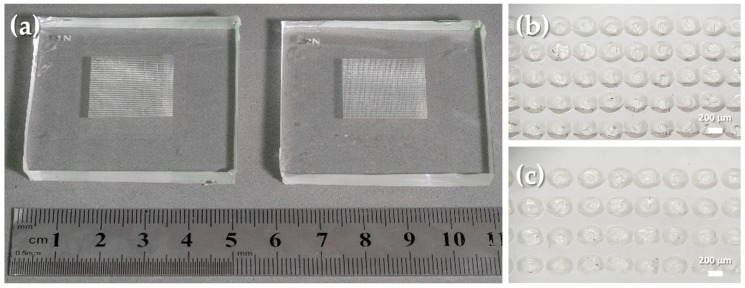
Hard PDMS molds engraved with circles with a diameter of 200 µm, at a laser power of 7.5 W and a laser speed of 0.254 m/s. (**a**) Photograph of the engraved molds. (**b**) Close-up view of the cavities of a mold engraved with circles with a pitch of 150 µm. (**c**) Close-up view of the cavities of a mold engraved with circles with a pitch of 200 µm.

After the optimization of the conditions to achieve semi-spheres, a design based on circles with a diameter of 200 μm and a pitch of 150 μm or 200 μm was engraved on h-PDMS molds with a laser power of 7.5 W and a laser speed of 0.254 m/s. The photographs of these molds are presented in Figure 4a. Figure 4b,c show a close-up view of the mold cavities engraved with a circle pitch of 150 μm and 200 μm, respectively, where one may perceive the regularity of the cavities’ shape, dimension, and pitch. 

### 3.2. Characterization of Coated s-PDMS Micro-Structured Films

**Figure 5:**
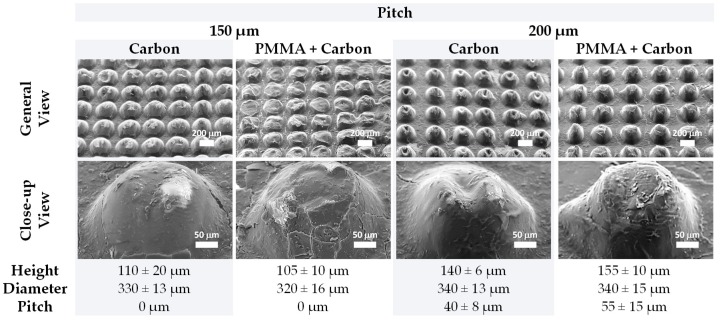
General view and close-up view of s-PDMS semi-spheres peeled off from optimized molds (circles with a diameter of 200 µm, engraved with a laser power of 7.5 W and a laser speed of 0.254 m/s) with different pitches and with carbon coating only or with both PMMA and carbon coating. Below the SEM images are displayed the measurements of the real height, diameter, and pitch of the semi-spheres. Note that the values correspond to average values ± standard deviation of a minimum of three measurements.

Using molds with the optimized parameters to obtain semi-sphere like structures (those shown in Figure 4), s-PDMS films were peeled off, with an average thickness of (215 ± 23) μm (for 16 measurements), and coated with carbon coating or both PMMA and carbon coating. Herein, the use of PMMA layer aimed at a greater adhesion of carbon coating to s-PDMS. SEM images were acquired to analyze the impact of the coated films on the micro-structure features, as shown in Figure 5. These general views of the semi-spheres (according to the vertical direction of the engraving process) highlight the good homogeneity of the structures and a perfect alignment over both the vertical and horizontal directions. When facing two films to produce a piezoresistive sensor, the pitch and diameters chosen for these patterns will promote the contact between both groups of semi-spheres, thereby granting reproducibility over sensors and possibly contributing to a lower nominal resistance due to the existence of more contact spots for the flowing of electrical current. Additionally, the real measurements of the semi-sphere features, namely height, diameter, and pitch, reveal that the thickness of the carbon coating and the PMMA films is small and has no impact on these features. In fact, the values of these features are very similar for films with PMMA and carbon coating or just carbon coating, being also very close to those of films without these coated films (Table 1). The close-up view of these semi-spheres also shows that the coated films completely cover them in a homogeneous way, which is important for the correct performance of the sensors. 

### 3.3. Electrical Characterization of E-Skin Piezoresistive Sensors

**Figure 6:**
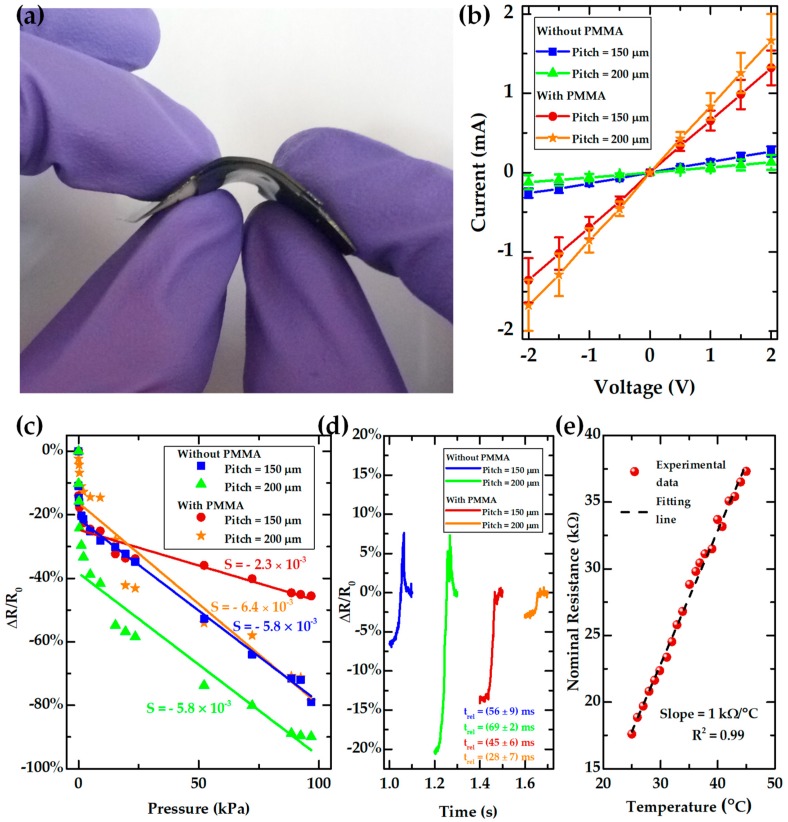
Electrical characterization of e-skin piezoresistive sensors with and without a PMMA layer, presenting a semi-sphere theoretical pitch of 150 µm or 200 µm. (**a**) Photography of one e-skin piezoresistive sensor under bending. (**b**) Current–voltage (I–V) curves of the sensors. (**c**) Relative resistance change of the sensors under a pressure of 79 Pa to 100 kPa. (**d**) Relaxation time of the sensors upon unloading of a small magnet (184 Pa). Note that the values correspond to average values ± standard deviation of seven measurements. (**e**) Nominal resistance of a sensor without a PMMA layer and with a semi-sphere theoretical pitch of 150 µm for a temperature range of 25 °C to 45 °C. The dashed black line corresponds to a linear regression with a slope of 1 kΩ/°C and a an *R*^2^ value of 0.99.

Figure 6a shows one e-skin piezoresistive sensor under bending to illustrate its flexibility. Figure 6b presents the I–V curves of the sensors produced with s-PDMS films peeled off from optimized molds, with semi-spheres having a theoretical pitch of 150 µm or 200 µm, with or without the PMMA layer. As expected, the sensors have an ohmic behavior. Additionally, sensors without the PMMA layer have a nominal resistance of 7.5 kΩ or 15.8 kΩ for a semi-sphere theoretical pitch of 150 µm or 200 µm, respectively. However, upon the presence of the PMMA layer, the nominal resistance of the sensors decreases to 1.5 kΩ or 1.2 kΩ for a semi-sphere theoretical pitch of 150 µm or 200 µm, respectively. Such results seem to point toward a contribution of PMMA to the electrical stability of the sensor. This effect is also corroborated by the fact that micro-structured s-PDMS films without PMMA present larger sheet resistance values with larger standard deviations than films with PMMA (Figure S1, Supplementary Materials), which may be attributed to a poorer adhesion of carbon coating directly to PDMS, thus translating into a greater electrical instability. Contact-angle measurements in PDMS films with and without the PMMA layer also show that, either before or after an oxygen plasma treatment to the films, the presence of PMMA decreases the contact angle, which makes the surface more hydrophilic and helps the adhesion of the water-based carbon coating to the films (Figure S2, Supplementary Materials). The nominal resistance values could be modified by changing the concentration of the carbon coating film, or by varying the thickness of the carbon coating, as it was already studied in previous work [46]. Nevertheless, the nominal resistance of the sensors should not be excessively high and their flexibility should be preserved. Therefore, a carbon coating thickness of approximately 3 µm with a sheet resistance of 1.3 kΩ/□ was used in these sensors as a compromise between nominal resistance and flexibility.

The sensitivity of the sensors was estimated through Equation (1).

(1) $$S=\frac{d\left(\frac{∆R}{R_{0}}\right)}{dP},$$

where ∆*R* is the resistance change, *R*_0_ is the initial resistance of the sensor when no pressure is being applied, and *P* is the compressive pressure. Figure 6c shows the relative resistance change of the sensors for each design for a pressure range from 79 Pa to 100 kPa. The respective sensitivity values for each sensor in a low-pressure range (from 0 Pa to 400 Pa) and a high-pressure range (from 1.2 kPa to 100 kPa) is presented in Table 2. Although there is not a clear trend for the sensitivity change with the presence or absence of the PMMA layer, sensors without the PMMA layer presented a more unstable performance upon the loading and unloading of weights than sensors with this layer (Figure S3, Supplementary Materials). In fact, sensors without PMMA layer did not always maintain an approximately constant nominal resistance under a fixed pressure, showing some sudden resistance changes that were associated with electrical instability of the carbon coating, since the sensors were not being stimulated. For sensors with the PMMA layer, their nominal resistance was kept constant during a fixed pressure, only showing sudden changes when the weight was unloaded. There is also no clear trend for the sensitivity change with the semi-sphere theoretical pitch; however, the results highlight the fact that the sensors have a very stable sensitivity value, as high as −6.4 × 10^−3^ kPa^−1^ throughout a long pressure range that falls between the relevant values for functional prosthesis and robotics, from less than 10 kPa to 100 kPa [20,28]. Table 3 summarizes the performance of several e-skin like sensors reported in the literature and compares them to the ones obtained herein, where one may notice that the sensitivity values reached in this work are 10 times better than that of some reported sensors [5,29,44], being also constant over a wider pressure range, while other sensors suffer a sensitivity decrease below 10 kPa [26,34,42]. Additionally, the majority of sensors reported so far were not tested in the full range of pressures that are relevant for these applications (10 kPa to 100 kPa), as shown in Table 3. Similar e-skin sensors to those presented herein, but having micro-structures with the shape of cones instead of semi-spheres, can reach higher sensitivities but in a reduced pressure range, with a maximum of −2.52 kPa^−1^ below 160 Pa, as this group already reported [46]. Such facts demonstrate that the laser engraving technique allows a great customization of the micro-structuration design, which has a direct impact in the performance of the sensors. Furthermore, the local defects that could be observed in the micro-structured s-PDMS films in Figure 3 did not hamper the sensing performance of the sensors, which highlights the high benefit/cost ratio of this micro-structuration technique. 

The relaxation time of each sensor was estimated by loading and unloading a small magnet (184 Pa) on top of them, as shown in Figure 6d. Sensors with the PMMA layer present slightly better relaxation times than sensors without the presence of such layer, reaching a low value of (28 ± 7) ms for a semi-sphere pitch of 200 µm. This value is better than some relaxation times reported for other sensors [9,10,26,42], as shown in Table 3. Furthermore, the presence of the PMMA layer seems to smooth the sensors’ behavior during relaxation, while sensors without PMMA have a peak before stabilizing their resistance. Such behavior may be explained by a contribution of PMMA for the reduction of the viscoelastic behavior that PDMS has when used alone [28,51].

Figure 6e shows the nominal resistance change of one sensor (without the PMMA layer and with semi-spheres with a theoretical pitch of 150 µm) with temperature. The data show that these pressure sensors may also be used to monitor temperature due to their excellent linearity between their nominal resistance in the absence of a pressure and the applied temperature in one side of the sensor, with an *R*^2^ value very close to 1 and a slope of 1 kΩ/°C. The temperature coefficient of resistance (TCR) may be estimated through Equation (2).

(2) $$TCR=\frac{\frac{∆R}{R_{0}}}{T-T_{0}},$$

where ∆*R* is the resistance change, *R*_0_ is the initial resistance of the sensor, *T* is the temperature at which the sensor was heated, and *T*_0_ is the initial temperature of the sensor. A TCR of 8.3%/°C was reached with the sensor tested in Figure 6e, which is quite high when compared to other reported values [37,43,44,45] as shown in Table 3. The detection of temperature changes by the sensor could be attributed to three effects: a sheet resistance change of the active material of the sensor (the carbon coating film) with the temperature, a thermal expansion of PDMS (with a coefficient of thermal expansion of 3 × 10^−4^ °C^−1^ [52]) that induces a stretching of the carbon coating, thus leading to an increase in the carbon coating sheet resistance, and a change in the contact resistance between the semi-spheres of the sensor (please consult Figure S4, Supplementary Materials, for further details). The estimation of the contribution of each effect to the final performance of the sensor is not trivial and would require the establishment of a complex mathematic model of the sensor. Nonetheless, the data shown validate this e-skin sensor as a sensitive temperature sensor within the range of human body temperatures.

**Figure 7:**
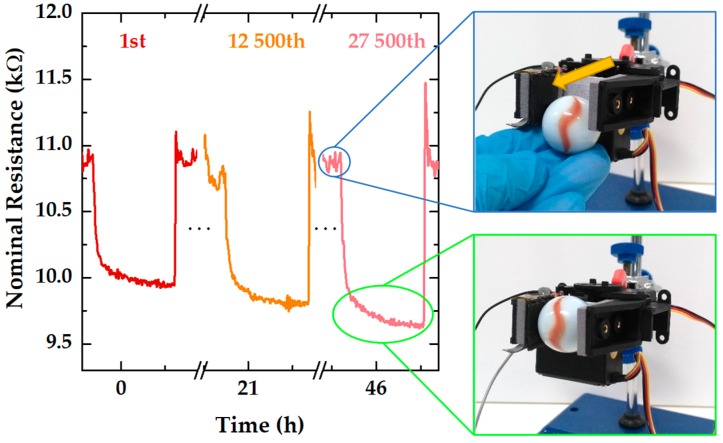
Resistance change for the cyclic grasping and releasing of an object by a robotic arm, from the first cycle to the 27,500th cycle, monitored by an e-skin piezoresistive sensor with a semi-sphere pitch of 150 µm and with the PMMA layer. This test was performed at 23 °C, fixed humidity of 60%, and a constant grasping pressure of approximately 160 Pa. The inset with a blue outline shows the robotic arm releasing an illustrative object. The inset with the green outline shows the robotic arm grasping the illustrative object. The sensor (marked with an orange arrow) is placed over the gray sponge of the robotic arm and has black and gray wires connecting it to the remaining readout equipment.

To test the performance of these sensors in a robotics application and to simultaneously perform a fatigue evaluation, a sensor was placed in a robotic arm to monitor the pressure change throughout the cyclic grasping and releasing of an object, at 23 °C, fixed humidity of 60%, and a constant grasping pressure of approximately 160 Pa. We opted to perform this test using a sensor with a PMMA layer and a semi-sphere theoretical pitch of 150 µm because sensors with PMMA have a more stable performance, which makes them better than sensors without such a layer. Figure 7 shows the cyclic change of the nominal resistance of the referred sensor placed in the robotic arm. When the robotic arm grasps the object, the nominal resistance of the sensor decreases (inset with the green outline) due to the pressure increase; however, after some seconds, the robotic arm releases the object and the resistance of the sensor goes up to its original value (inset with the blue outline). A resistance peak shows up when the robotic arm releases the object due to adhesion forces between the sensor and the object. After 12,500 cycles (corresponding to almost 21 h of continuous motion) or even 27,500 cycles (corresponding to almost 46 h of continuous motion), the resistance change of the sensor does not show significant differences when compared to the first grasping and releasing cycle. SEM images before and after the fatigue test show no evident signs of the impact of such a high number of pressure cycles on the micro-structured films (Figure S5, Supplementary Materials). These data point toward a highly stable performance of the sensor. Such stability, summed to fast relaxation times, a constant sensitivity from 1.2 kPa to 100 kPa, and a high TCR, show that these e-skin piezoresistive sensors have great potential for applications in the robotics and functional prosthesis fields, where the detection of a large spectrum of pressures is required and a cyclic detection also plays a crucial role. 

## 4. Conclusions

The present work demonstrates the versatility and high benefit/cost ratio of the laser engraving technique for the production of h-PDMS molds, used to micro-structure s-PDMS films that compose bimodal e-skin piezoresistive and temperature sensors. Through the micro-structuration of such films into semi-spheres, instead of micro-cones as explored in a previous work, and with the presence of a PMMA layer that is crucial for the electrical stability of the sensors, the sensitivity of the sensors (with a maximum value of −6.4 × 10^−3^ kPa^−1^) could be kept constant in a large pressure range that is important for prosthesis and robotics applications, from 1.2 kPa to 100 kPa. With a highly stable performance over 27,500 cycles and a high TCR (8.3%/°C), these semi-sphere sensors are promising for this type of applications and contrast with some sensors reported so far that either use expensive fabrication techniques to achieve similar performances, or are not tested in the full range of pressures that are meaningful for robotics and functional prosthesis. 

## Figures and Tables

**Table 1:** Measurements of real diameter and pitch for standard polydimethylsiloxane (s-PDMS) micro-structures peeled off from hard PDMS (h-PDMS) molds engraved with circles with different diameters, different pitches, with a laser power of 7.5 W and a laser speed of 0.254 m/s. These measurements were performed either according to the horizontal direction or the vertical direction of the engraving process. The theoretical sum values correspond to the sum of the theoretical pitch and the theoretical diameter, while the real sum values correspond to the sum of the real pitch and the real diameter. The relative difference is related to the difference between the real sum and the theoretical sum. Note that the values correspond to average values of a minimum of 15 measurements.

Power = 7.5 W								
Laser Engraving Direction	Horizontal				Vertical			
Theoretical Pitch (µm)	150	150	200	200	150	150	200	200
Theoretical Diameter (µm)	100	200	100	200	100	200	100	200
Theoretical Sum (µm)	250	350	300	400	250	350	300	400
Real Pitch (µm)	0	23	43	37	63	63	103	95
Real Diameter (µm)	240	322	260	350	183	277	186	280
Real Sum (µm)	240	345	303	387	246	339	289	375
Relative Difference	−4.0%	−1.3%	0.9%	−3.3%	−1.4%	−3.0%	−3.7%	−6.3%

**Table 2:** Sensitivities of the sensors shown in Figure 6c for the low-pressure range (from 0 Pa to 400 Pa) and the high-pressure range (from 1.2 kPa to 100 kPa).

		Without PMMA		With PMMA	
Theoretical Pitch (µm)		150	200	150	200
Low Pressure (0 kPa–0.4 kPa)	Sensitivity (kPa^−1^)	−4.8 × 10^−1^	−6.4 × 10^−1^	−5.4 × 10^−1^	−1.8 × 10^−1^
High Pressure (1.2 kPa–100 kPa)	Sensitivity (kPa^−1^)	−5.8 × 10^−3^	−5.8 × 10^−3^	−2.3 × 10^−3^	−6.4 × 10^−3^

**Table 3:** Performance of relevant e-skin sensors reported so far. Abbreviations: NA—not available; TCR—temperature coefficient of resistance.

Reference	Transduction Mechanism	Micro-Structuration Process and Shape	Pressure Sensitivity and Range	Maximum Pressure Tested	Relaxation Time	Number of Pressure Cycles	TCR and Range
[5]	Capacity	-	5 × 10^−4^ kPa^−1^ (<1 MPa)	1 MPa	NA	NA	-
[6]	Capacity	Photolitography Pyramids	8.4 kPa^−1^ (<8 kPa) 0.38 kPa^−1^ (>30 kPa)	~60 kPa	10 ms	15,000	-
[10]	Capacity	Photolitography Fibers	0.58 kPa^−1^ (<0.5 kPa)	10 kPa	30 ms	3000	-
[29]	Capacity	-	2.3 × 10^−4^ kPa^-1^ (<0.8 MPa)	800 kPa	NA	NA	-
[44]	Capacity	-	(3.4–5) × 10^−2^ kPa^−1^ (<0.1 kPa) 5 × 10^−4^ kPa^−1^ (10 kPa–25 kPa)	25 kPa	NA	2000	~ 0.2%/°C (25–50 °C)
[34]	Triboelectricity	Photolitography Pyramids	0.31 kPa^−1^ (<3 kPa) 1 × 10^−2^ kPa^−1^ (3 kPa–13 kPa)	90 kPa	5 ms	30,000	-
[9]	Piezoresistivity	Photolitography Domes	−15.1 kPa^−1^ (<0.5 kPa)	59 kPa	40 ms	1000	-
[26]	Piezoresistivity	Photolitography Domes	14 kPa^−1^ (<5 kPa) 3.2 kPa^-1^ (5 kPa–10 kPa)	12 kPa	30 ms	10,000	-
[37]	Piezoresistivity	Photolitography Pyramids	2.5 kPa^−1^ (<250 Pa)	5 kPa	20 ms	100,000	0.32%/°C (40–43 °C)
[42]	Piezoresistivity	Using nylon as mold Cubic-like structures	56.36 kPa^−1^ (<1 kPa) 2.51 kPa^−1^ (1 kPa–10 kPa)	10 kPa	300 ms	25,000	-
[43]	Piezoresistivity	Photolitography Domes	NA	49.5 kPa	NA	5000	2.93%/°C (20–40 °C)
[45]	Piezoresistivity	Stretching Wrinfles	NA	NA	NA	300	2.38%/°C (30–65 °C)
**This work**	Piezoresistivity	Laser engraving Domes	−(1.8–6.4) × 10^−1^ kPa^−1^ (<400 Pa) −(2.3–6.4) × 10^−3^ kPa^−1^ (1.2 kPa–100 kPa)	100 kPa	(28 ± 7) ms	27,500	8.3%/°C (25–45 °C)

## Supplementary Materials

The following are available online at https://www.mdpi.com/1424-8220/19/4/899/s1, Figure S1: Sheet resistance of carbon coating on micro-structured s-PDMS films. Note that the values represented correspond to average values ± standard deviation of a minimum of 3 films, Figure S2: Contact angle for PDMS films and PDMS films coated with PMMA, before and after being subjected to an oxygen plasma treatment. Note that the values represented correspond to average values ± standard deviation of a minimum of 6 measurements. For PDMS films coated with PMMA, the contact angle after the oxygen plasma treatment is represented as being 10°, however, the real value is smaller but it cannot be precisely estimated due to technique limitations, Figure S3: Resistance change when removing weights from the top of sensors. (**a**) Resistance change from 20 kPa to 15 kPa of a sensor with a PMMA layer and a semi-spheres theoretical pitch of 150 μm. (**b**) Resistance change from 26 Pa to 0 Pa of a sensor without a PMMA layer and with a semi-spheres theoretical pitch of 200 μm, Figure S4: Resistance change of different samples with temperature, for a temperature range from 25 °C and 45 °C. (**a**) Sheet resistance change of carbon coating spin-coated on a glass. The dashed black line corresponds to a linear regression with a slope of 0.87 Ω/°C and a R2 of 0.90. (**b**) Sheet resistance of a semi-spheres s-PDMS film (pitch of 150 μm) coated with carbon coating. The dashed black line corresponds to a linear regression with a slope of 0.24 kΩ/°C and a R2 of 0.99. (**c**) Nominal resistance of a sensor without PMMA layer and with a semi-spheres theoretical pitch of 150 μm. The dashed black line corresponds to a linear regression with a slope of 1 kΩ/°C and a R2 of 0.99, Figure S5: SEM images of s-PDMS films with PMMA and carbon coating, peeled off from optimized molds (circles with a diameter of 200 μm, engraved with a laser power of 7.5 W and a laser speed of 0.254 m/s). (**a**) General view of a film before applying cyclic pressure. (**b**) Close-up view of a film before applying cyclic pressure. (**c**) General view of another film after applying cyclic pressure (27500 cycles of grasping and releasing of an object). (**d**) Close-up view of another film after applying cyclic pressure (27500 cycles of grasping and releasing of an object).
