# Supplementary material for: E-Skin Bimodal Sensors for Robotics and Prosthesis Using PDMS Molds Engraved by Laser
Source: Sensors (Basel). 2019 Feb 21;19(4):899. doi: 10.3390/s19040899 (PMC6412975; doi:10.3390/s19040899)
Supplement: Supplementary file 1 [file sensors-19-00899-s001.pdf]

sensors

Supplementary Information

E-skin Bimodal Sensors for Robotics and Prosthesis using PDMS Molds Engraved by Laser

Andreia dos Santos, Nuno Pinela, Pedro Alves, Rodrigo Santos, Ricardo Farinha, Elvira Fortunato, Rodrigo Martins, Hugo Águas and Rui Igreja*

CENIMAT|i3N, Departamento de Ciência dos Materiais, Faculdade de Ciências e Tecnologia, Universidade Nova de Lisboa, Campus de Caparica, 2829-516 Caparica, Portugal; ass.santos@campus.fct.unl.pt (A.d.S.); n.pinela@campus.fct.unl.pt (N.P.); pedro.alves@strath.ac.uk (P.A.); rcd.santos@campus.fct.unl.pt (R.S.); rd.farinha@campus.fct.unl.pt (R. F.); emf@fct.unl.pt (E.F.); rm@uninova.pt (R.M.); hma@fct.unl.pt (H.A.)

* Correspondence: rni@fct.unl.pt; Tel.: +351 21-294 8562

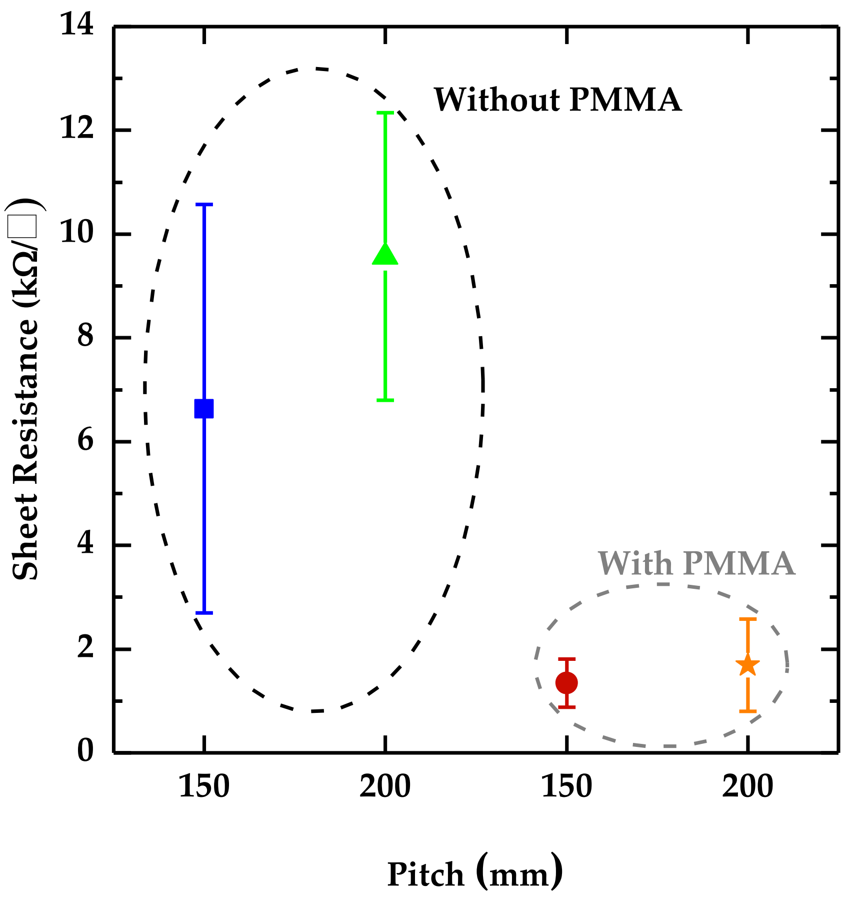

Figure S1. Sheet resistance of carbon coating on micro-structured s-PDMS films. Note that the values represented correspond to average values ± standard deviation of a minimum of 3 films.

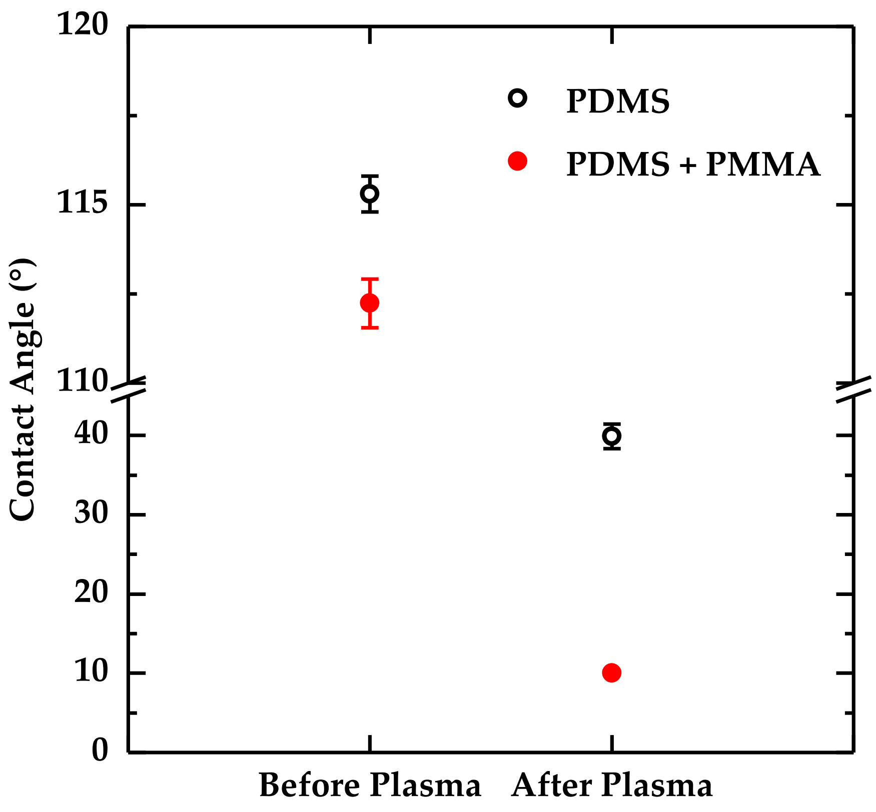

Figure S2. Contact angle for PDMS films and PDMS films coated with PMMA, before and after being subjected to an oxygen plasma treatment. Note that the values represented correspond to average values ± standard deviation of a minimum of 6 measurements. For PDMS films coated with PMMA, the contact angle after the oxygen plasma treatment is represented as being 10°, however, the real value is smaller but it cannot be precisely estimated due to technique limitations.

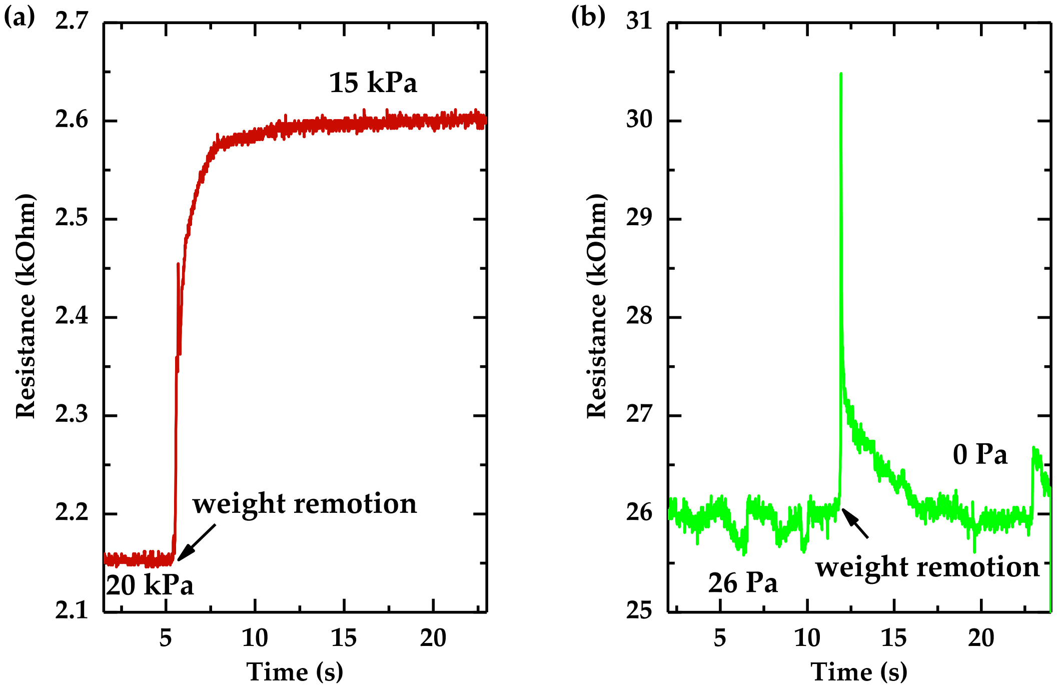

Figure S3. Resistance change when removing weights from the top of sensors. (**a**) Resistance change from 20 kPa to 15 kPa of a sensor with a PMMA layer and a semi-spheres theoretical pitch of 150 µm. (**b**) Resistance change from 26 Pa to 0 Pa of a sensor without a PMMA layer and with a semi-spheres theoretical pitch of 200 µm.

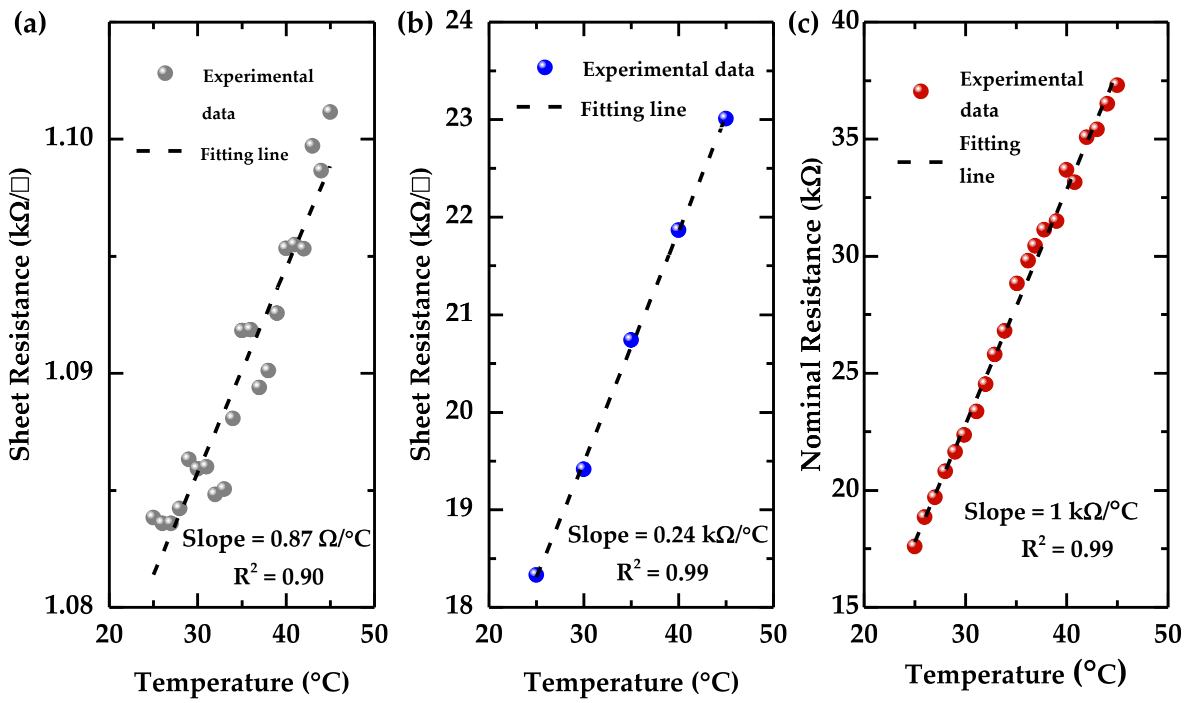

Figure S4. Resistance change of different samples with temperature, for a temperature range from 25 °C and 45 °C. (**a**) Sheet resistance change of carbon coating spin-coated on a glass. The dashed black line corresponds to a linear regression with a slope of 0.87 Ω/°C and a R^2 of 0.90. (**b**) Sheet resistance of a semi-spheres s-PDMS film (pitch of 150 μm) coated with carbon coating. The dashed black line corresponds to a linear regression with a slope of 0.24 kΩ/°C and a R^2 of 0.99. (**c**) Nominal resistance of a sensor without PMMA layer and with a semi-spheres theoretical pitch of 150 μm. The dashed black line corresponds to a linear regression with a slope of 1 kΩ/°C and a R^2 of 0.99.

As it was mentioned in the article, there are some effects that contribute to the performance of the e-skin sensor as a temperature sensor, namely:

1. A sheet resistance change of the sensor's active material with temperature. In this case, the active material is carbon coating;
2. A thermal expansion of s-PDMS. PDMS has a high coefficient of thermal expansion of 3×10^{-4} °C^{-1} [1] and so, upon warming, PDMS expands and induces the stretching of the carbon coating film, consequently leading to an increase of this film's sheet resistance with temperature;
3. A change in the contact resistance between the semi-spheres of the sensor.

In an attempt to estimate the contribution of each effect to the performance of the sensor, the following studies were performed:

1. For the contribution of only carbon coating, this material was spin coated on glass, a substrate that has a low coefficient of thermal expansion of 0.5×10^{-6} °C^{-1} [2] to avoid the interference of the substrate's expansion. The change of sheet resistance of this film with temperature was then measured, as shown in Figure S4(a), and a TCR of 0.080 %/°C was obtained.
2. For the contribution of s-PDMS thermal expansion, carbon coating was spin-coated on a s-PDMS film with micro-spheres with a theoretical pitch of 150 μm. The change of sheet resistance of this micro-structured film with temperature was then measured, as shown in Figure S4(b), and a TCR of 1.28 %/°C was obtained. Nevertheless, this value already accounts for the contribution of the carbon coating film itself, given that we could not measure the sheet resistance of a PDMS film without the active film through the setup we used to perform the temperature measurements.

3. The contribution of the contact resistance change is hard to experimentally estimate.

Given that the TCRs obtained for each effect are smaller than the TCR for the whole sensor, we foresee that the contact resistance effect also plays a relevant role in the performance of the sensor as a temperature sensor. However, it is complex to understand how each effect may affect each other. To better understand these effects, their relation and impact, and possibly estimate a theoretical contribution of each one in the final performance of the sensor, a mathematical model of the sensor is currently under development.

References:

1. Grzybowski, B. A.; Brittain, S. T.; Whitesides, G. M. Thermally actuated interferometric sensors based on the thermal expansion of transparent elastomeric media. *Rev. Sci. Instrum.* **1999**, *70*, 2031–2037, doi:10.1063/1.1149706.
2. Roy, R.; Agrawal, D. K.; McKinstry, H. A. Very Low Thermal Expansion Coefficient Materials. *Annu. Rev. Mater. Sci.* **1989**, *19*, 59–81, doi:10.1146/annurev.ms.19.080189.000423.

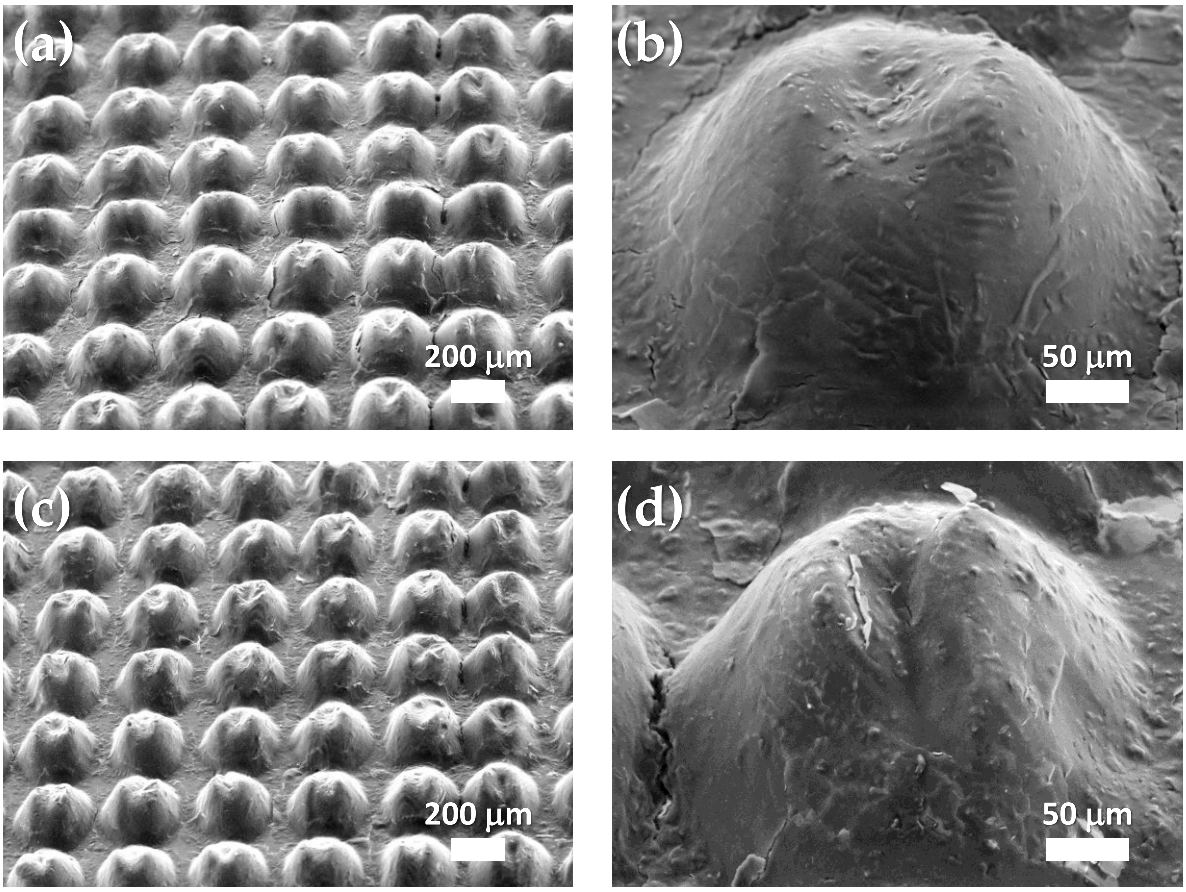

Figure S5. SEM images of s-PDMS films with PMMA and carbon coating, peeled off from optimized molds (circles with a diameter of 200 μm, engraved with a laser power of 7.5 W and a laser speed of 0.254 m/s). (**a**) General view of a film before applying cyclic pressure. (**b**) Close-up view of a film before applying cyclic pressure. (**c**) General view of another film after applying cyclic pressure (27500 cycles of grasping and releasing of an object). (**d**) Close-up view of another film after applying cyclic pressure (27500 cycles of grasping and releasing of an object).
